# Supplementary material for: The partial mitochondrial genome of Semidalis anchoroides Liu & Yang, 1993 (Neuroptera: Coniopterygidae)
Source: Mitochondrial DNA B Resour. 2024 Nov 22;9(11):1592–5. doi: 10.1080/23802359.2024.2429642 (PMC11587714; doi:10.1080/23802359.2024.2429642)
Supplement: supplement meterial.docx [file TMDN_A_2429642_SM7695.docx]

Supplemental material


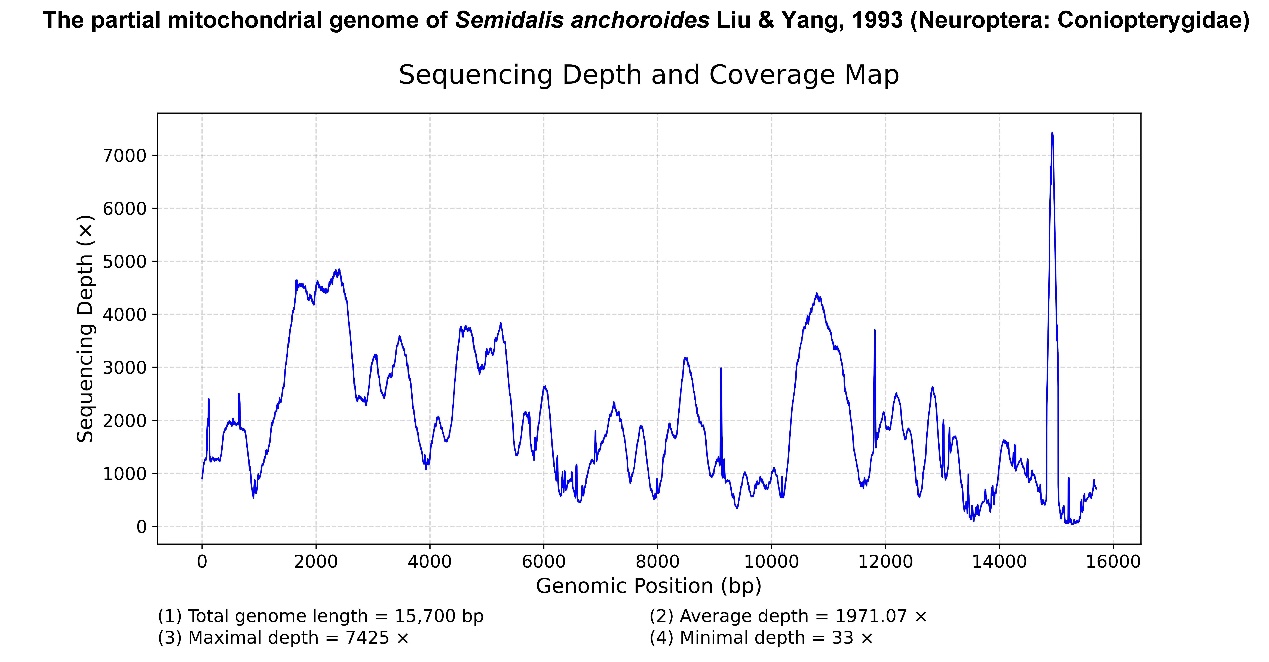


Supplemental Figure S1. Coverage depth plot of the assembled mitochondrial genome of *Semidalis anchoroides* (GenBank accession no. PP657419). The horizontal axis represents the nucleotide position, and the vertical axis represents the read mapping depth.
