# Supplementary material for: The partial mitochondrial genome of Semidalis anchoroides Liu & Yang, 1993 (Neuroptera: Coniopterygidae)
Source: Mitochondrial DNA B Resour. 2024 Nov 22;9(11):1592–5. doi: 10.1080/23802359.2024.2429642 (PMC11587714; doi:10.1080/23802359.2024.2429642)
Supplement: a clean copy of the manuscript.docx [file TMDN_A_2429642_SM7694.docx]

**The partial mitochondrial genome of** ***Semidalis anchoroides* Liu & Yang, 1993 (****Neuroptera: Coniopterygidae)**

Yaru Zhao^1, 2^, Yanyu Zhang^1^, Guangshuai Liu^1^, Ying Li^2^, Zhiqi Liu^2^*

1 School of Grain Science and Technology, Jiangsu University of Science and Technology, Zhenjiang 212004, China

2 Department of Entomology, China Agricultural University, Beijing 100094, China

*To whom the correspondence and reprint requests should be addressed: [liuzhiqi@cau.edu.cn](mailto:liuzhiqi@cau.edu.cn)

**Abstract**

*Semidalis anchoroides* Liu & Yang, 1993 is a small and common insect in southern China. It’s known for its small sized, being covered by whitewax powder, and having uncomplicated venation. In this study, the mitochondrial genome for *S. anchoroides* was sequenced and analyzed. The sequenced partial mitogenome is 15700 bp in length, encoding 13 protein-coding genes (PCGs), 22 tRNA genes, 2 rRNA genes, and 1 partial control region. These findings provide fundamental molecular data, thereby facilitating a more profound understanding of the phylogenetic relationships within the *Semidalis* species.

**Keywords** dustywings; mitogenome; phylogenetic

Introduction

The Coniopterygidae family, comprising over 570 species, is recognized as the smallest and most unique group within the order Neuroptera (Sziráki 2011; Engel et al. 2018). Commonly known as “dustywings”, this lineage exhibits a broad distribution, with the exception of extremely cold regions. It is particularly atypical among lacewings for its small size, the distinctive white wax powder (or sometimes light greyish or yellowish) that covers its body, and its simple wing venation (Meinander 1972). The genus *Semidalis* possesses approximately 75 species, with 13 species in China (Zhao et al., 2021). *Semidalis anchoroides* Liu & Yang, 1993 is widely distributed in the south of China, such as Yunnan, Guangzhou, Guizhou.

The mitochondrial genome has been widely used to resolve phylogenetic relationships among lacewings (Wang et al., 2017). To date, mitochondrial genomes of only three species in Coniopterygidae have been sequenced (Wang et al., 2017; Song et al., 2019). In this study, we reported the partial mitochondrial genome (mitogenome) of *S. anchoroides* and reconstruct the phylogenetic relationship using the current mitogenome data from these three species of Coniopterygidae, as well as data from five other families within the order Neuroptera.

Materials


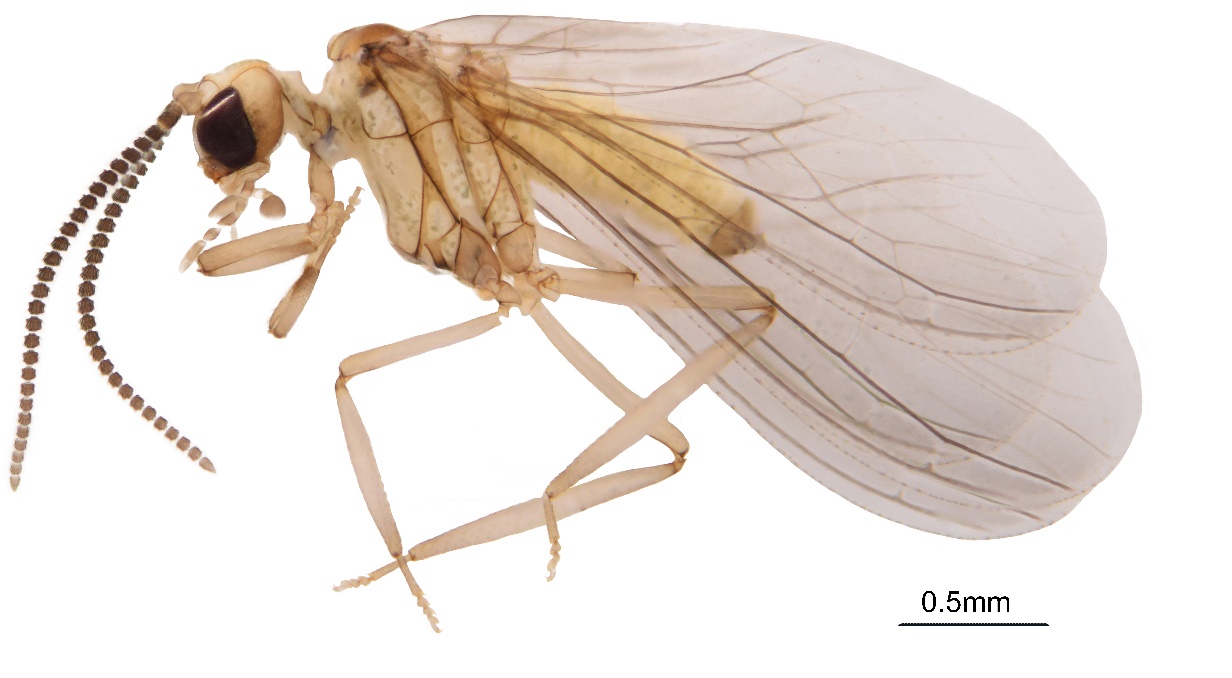


Figure 1. Morphology of *Semidalis anchoroides*. (photo by Ying Li, unpublished). The species generally covered by whitewax powder (or sometimes light greyish or yellowish), and the venation is uncomplicated.

The *S. anchoroides* specimen used in this study was obtained from Longchuan forest Park in Ruili City of Yunnan Province in March 2019 (24.1776N, 97.7947E) by Yaru Zhao & Mingming Zou (Figure 1). Morphological identification was based on the following taxonomic characteristics: there are no plicatures on the abdominal sternites, vein M is forked in the hind wing, vein Rs is forked in both wings, the cross-vein M-Cu1 of both wings is oblique and it strikes the branch M3+4 or the fork of M, parameres have one dorsal knob, and uncini are present and not fused (Meinander 1972; Sziráki 2011). Voucher specimen has been deposited at the Entomological Museum of China Agricultural University (https://cpp.cau.edu.cn/, Liu ZQ, [liuzhiqi@cau.edu.cn](mailto:liuzhiqi@cau.edu.cn)) under the voucher number CAU573. Photos were taken with a Nikon D5300 digital camera attached to a Leica DM2500 stereomicroscope. The resulting images were edited and processed with Adobe Photoshop CC 2018.

Methods

The total genomic DNA was extracted from thoracic muscle tissues using the TIANamp Genomic DNA Kit (Tiangen Biotech, Beijing, China) following the manufacturer’s instructions. The methods for analyzing the mitochondrial genome primarily referenced those of Xu et al. (2020). An Illumina TruSeq library, designed with an insert size of 350 base pairs (bp), was sequenced on the Illumina HiSeq 250 platform (Berry Genomics, Beijing, China) using 150 bp paired-end reads. After the removal of adapters and low-quality sequences with Trimmomatic-0.38 (Bolger et al. [2014](https://www.tandfonline.com/doi/full/10.1080/23802359.2024.2385610?src=exp-la)), we obtained 6Gb of clean and high-quality reads. These reads were then utilized for de novo assembly with the IDBA-UD software Peng et al. (2011). To accurately identify the mitochondrial (mt) genome sequences, the assembled contigs were cross-referenced against the cytochrome oxidase I (*COI*) gene sequences of this species using BLAST, with a similarity threshold set at a minimum of 98% (Simon et al. 2006). The resultant mitogenome sequence is linear and incomplete. We failed to recover the complete mitogenome through Sanger sequencing. The sequenced data were annotated using tRNAscan-SE (Lowe & Eddy, 1997), Mitos (Bernt et al. 2013), and Geneious v10.2.6 (Biomatters Ltd., Auckland, New Zealand). The methods for analyzing the sequencing depth and coverage map primarily referenced those of Ni et al. (2023).

For phylogenetic analyses, we used the mitogenome sequences of seven species in the order Neuroptera, which were downloaded from the GenBank database (Table 1). *Chrysopa pallens* (Rambur, 1838) was selected as the outgroup. All mitogenomes of Coniopterygidae were used in GenBank. Nucleotide sequences of the 13 protein-coding genes (PCGs) were translated into amino acids and aligned individually using MAFFT (Katoh & Standley 2013) and MACSE (Ranwez et al. 2018) within PhyloSuite v1.2.2 (Zhang et al. 2020). The aligned sequences were trimmed by Gblocks (Talavera & Castresana [2007](https://onlinelibrary.wiley.com/doi/full/10.1111/1755-0998.13096#men13096-bib-0022)). Then the trimmed aligned sequences were concatenated. The concatenated dataset was used to construct a phylogenetic tree with the IQ-TREE (Nguyen et al. 2015) method, applying 1000 bootstrap replicates, facilitated by PhyloSuite v1.2.2. (Xiang et al. 2023; Zhang et al. 2020).

Results

The *S. anchoroides* partial mitogenome (GenBank No. PP657419) is 15,700 bp in length and consists of 13 PCGs, 22 tRNA genes, two rRNA genes, and a control region (Figure 2). The minimal and average coverage depths of this sequences are 33× and 1971.07×, respectively (Supplemental Figure S1). The minimal and average coverage depths of this sequences are 33× and 1971.07×, respectively (Supplemental Figure S1). The content of adenine, guanine, cytosine, and thymine in the mitogenome are 40.93%, 8.74%, 12.21% and 38.12%, respectively. Among 13 PCGs, the start codon is ATN, the stop codon is TAA (CO*X1*, CO*X2*, *COX3*, and *ND5* (T)). Nine PCGs (*ND3*, *COX3*, *ATP6*, *ATP8*, CO*X2*, *COX1*, *ND2*, *CytB*, *ND6*) and 14 tRNA (*trnI,* *trnM*, *trnW*, *trnL_2_*, *trnK,* *trnD*, *trnG*, *trnA*, *trnR*, *trnN*, *trnS_1_*, *trnE*, *trnT*, *trnS_2_*,) genes are encoded on the heavy strand (H-strand), and the remaining genes are located on the light strand (L-strand).

The phylogenetic analysis is shown in Figure 3 based on 13 PCGs by the IQ-tree method, with *C. pallens* used as an outgroup. The mitogenome of *S. anchoroides* is a sister to the mitogenome of its congener (*S. aleyrodiformis*) with 100% support. In addition, the mitogenome of they and *S. macleodi* cluster a clade with 100% support. And the mitogenome of all Coniopterygidae and Coniopteryginae form a clade, respectively.


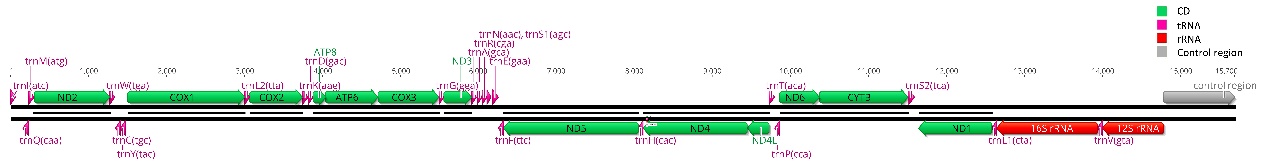


Figure 2. Mitogenome map of *S. anchoroides*. 37 genes were predicted, including 13 protein-coding genes (green bar), 22 tRNA genes (magenta bar), two rRNA genes (red bar) and one control region (grey bar). This plot was produced in Geneious (version 2024.0.5).


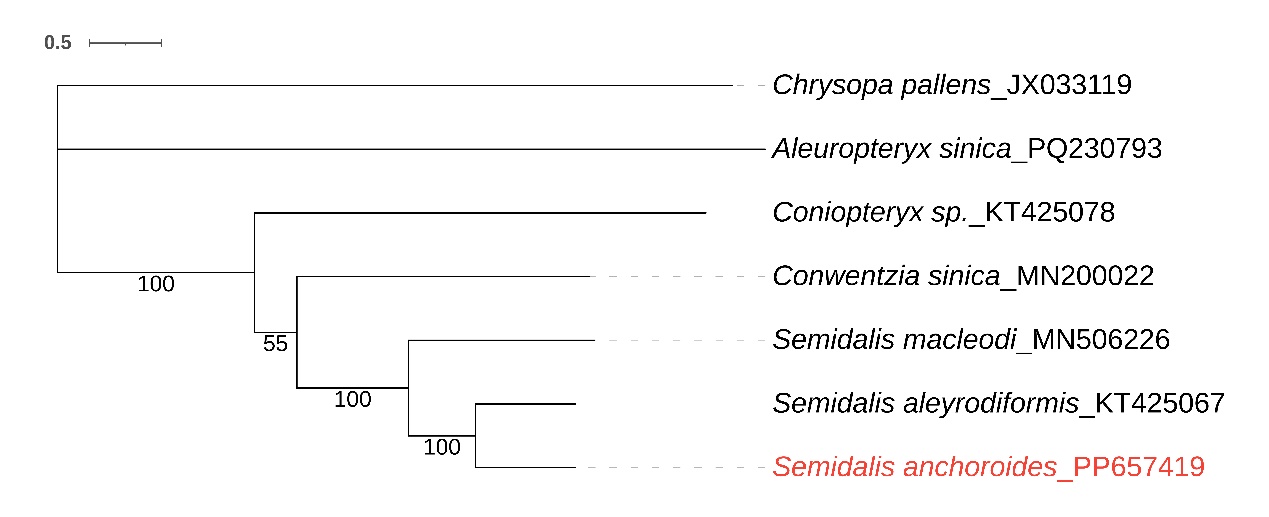


Figure 3. Phylogenetic trees of *S. anchoroides* with five dustywings species based on 13 PCGs by IQ-tree method. *C. pallens* was selected as the outgroup. The sequences used are listed in Table 1. The scale bar indicates the number of substitutions per site.

Discussion and conclusion

The partial mitogenome sequences of *S. anchoroides* are sequenced and annotated. There are no differences in gene number or arrangements between *S. anchoroides* and other dustywings (Song et al., 2019), which is retaining the putative ancestral insect mitogenome order (Engel et al. 2018). The partial mitochondrial genome of *Semidalis anchoroides* exceeds the length of its congeneric reference species due to variation in the length of the control region. The phylogenetic analysis indicates that the *Conwentzia* is the sister genus to the *Semidalis*, which is similar to the results of Meinander et al. (1972). This study enriches the mitochondrial genetic data of *S. anchoroides* to explore the phylogenetic relationships of dustywings.

References

Bernt A, Donath A, Jühling F, Externbrink F, Florentz C, Fritzsch G, Pütz J, Middendorf M, Stadler PF. 2013. MITOS: improved de novo metazoan mitochondrial genome annotation. Mol Phylogenet Evol. 69(2):313–319. doi.org/10.1016/j.ympev.2012.08.023

Bolger AM, Lohse M, Usadel B. 2014. Trimmomatic: a flexible trimmer for Illumina sequence data. Bioinformatics. [30](https://doi.org/10.1093/bioinformatics/btu170)([15](https://doi.org/10.1093/bioinformatics/btu170)):2114–2120. doi:10.1093/bioinformatics/btu170.

Engel MS, Winterton SL, Breitkreuz LCV. 2018. Phylogeny and evolution of Neuropterida: where have wings of lace taken us? Annu Rev of Entomol. 63:531–551. doi:10.1146/annurev-ento-020117-043127.

He K, Chen Z, Yu DN, Zhang JY. 2012. The complete mitochondrial genome of *Chrysopa pallens* (Insecta, Neuroptera, Chrysopidae). Mitochondrial DNA. 23(5):373–375. doi:10.3109/19401736.2012.696631.

Katoh K, Standley DM. 2013. MAFFT multiple sequence alignment software version 7: Improvements in performance and usability. Mol Biol Evol. 30(4):772–780. doi:10.1093/molbev/mst010.

Lowe TM, Eddy SR. 1997. tRNAscan-SE: a program for improved detection of transfer RNA genes in genomic sequence. Nucleic Acids Res. 25(5):955–964. doi:10.1093/nar/25.5.955.

Meinander M. 1972. A revision of the family Coniopterygidae (Planipennia). Acta Zool Fennica. 136:1–357. doi:10.5555/19720502775.

Nguyen LT, Schmidt HA, von Haeseler A, Minh BQ. 2015. IQ-TREE: a fast and effective stochastic algorithm for estimating maximum-likelihood phylogenies. Mol Biol Evol. 32(1):268–274. doi: 10.1093/molbev/msu300.

Ni Y, Li J, Zhang C, Liu C. 2023. Generating Sequencing Depth and Coverage Map for Organelle Genomes. doi:10.17504/protocols.io.4r3l27jkxg1y/v1.

Peng Y, Leung HCM, Yiu SM, Chin FYL. 2011. Meta-IDBA: a de Novo assembler for metagenomic data. Bioinformatics. 27(13):94–101. [doi:10.1093/bioinformatics/btr216](https://doi.org/10.1093/bioinformatics/btr216).

Ranwez V, Douzery EJP, Cambon C, Chantret N, Delsuc F. 2018. MACSE v2: Toolkit for the alignment of coding sequences accounting for frameshifts and stop codons. Mol Biol Evol. 35(10):2582–2584. doi:10.1093/molbev/msy159.

Simon C, Buckley TR, Frati F, Stewart JB, Beckenbach AT. 2006. Incorporating molecular evolution into phylogenetic analysis, and a new compilation of conserved polymerase chain reaction primers for animal mitochondrial DNA. Annu. Rev. Ecol. Evol. Syst. 37:545–579. doi:10.1146/annurev.ecolsys.37.091305.110018.

Song JL, Dong JY, Ma MW, He Y, Liu ZQ. 2019. The complete mitochondrial genome of *Conwentzia sinica* (Neuroptera: Coniopterygidae). Mitochondrial DNA Part B Resour. 4(2):4045–4046. doi:10.1080/23802359.2019.1688714.

Sziráki G. 2011. Coniopterygidae of the world: Annotated check-list and identification keys for living species, species groups and supraspecific taxa of the family. LAP Lambert Academic Publishing. 49–88.

Talavera G, Castresana J. 2007. Improvement of phylogenies after removing divergent and ambiguously aligned blocks from protein sequence alignments. Syst Biol. 56(4):564–577. doi:10.1080/10635150701472164.

Wang YY, Liu XY, Garzón-Orduña IJ, Winterton SL, Yan Y, Aspöck U, Aspöck H, Yang D. 2017. Mitochondrial phylogenomics illuminates the evolutionary history of Neuropterida. Cladistics. 33(6):617–636. doi:10.1111/cla.12186.

Xiang CY, Gao FL, Jakovlić I, Lei HP, Hu Y, Zhang H, Zou H, Wang GT, Zhang D. 2023. Using PhyloSuite for molecular phylogeny and tree-based analyses. IMeta. 2(1):e87. doi:https://doi.org/10.1002/imt2.87.

Xu H, Wu YF, Wang YJ, Liu ZQ. 2020. Comparative analysis of five mitogenomes of Osmylinae (Neuroptera: Osmylidae) and their phylogenetic implications. Int J Biol Macromol, 164:447–455. doi:10.1016/j.ijbiomac.2020.07.150.

Zhang D, Gao F, Jakovlić I, Zou H, Zhang J, Li WX, Wang GT. 2020. PhyloSuite: An integrated and scalable desktop platform for streamlined molecular sequence data management and evolutionary phylogenetics studies. Mol Ecol Resour. 20(1):348–355. doi:https://doi.org/10.1111/1755-0998.13096.

Zhao YR, Li Y, Li M, Liu ZQ. 2021. Two new species of *Semidalis* Enderlein, 1905 (Neuroptera, Coniopterygidae) from China, with an identification key to Chinese species. ZooKeys. 1055:43–54. doi:10.3897/zookeys.1055.63192.

Ethical approval

The material covered in the article does not involve any ethical conflict. This species is not endangered or collected in nature reserves, so it did not need any specific permissions. All acquisition and sequencing work were carried out in strict compliance with relevant local laws and laboratory regulations in order to preserve wild resources.

Authors’ contributions

Yaru Zhao and Zhiqi Liu conceived the project, Yaru Zhao, YanYu Zhang, GuangShuai Liu and Ying Li conducted experiments, Yaru Zhao and Zhiqi Liu wrote the manuscript. All authors agree to be accountable for all aspects of the work.

Table 1. Species and GenBank accession number of mitogenomes used in this study.

| Species | Accession ID | References |
| --- | --- | --- |
| *Chrysopa pallens* | JX033119 | He et al. 2012 |
| *Aleuropteryx sinica* | PQ230793 | Unpublished |
| *Coniopteryx sp.* | KT425078 | Wang et al. 2017 |
| *Conwentzia sinica* | MN200022 | Song et al. 2019 |
| *Semidalis macleodi* | MN506226 | Unpublished |
| *Semidalis aleyrodiformis* | KT425067 | Wang et al. 2017 |
| *Semidalis anchoroides* | PP657419 | In this study |

Disclosure statement

No potential conflict of interest was reported by the author(s).

Funding

This study was supported by the National Natural Science Foundation of China [No. 31772499].

Data availability statement

The genome sequence data supporting this study’s findings are openly available in GenBank of NCBI at https://www.ncbi.nlm.nih.gov with the accession number PP657419. The associated BioProject, SRA, and Bio-Sample numbers are PRJNA1115984, SRR29188912, and SAMN41519342, respectively.

Additional information

Funding

This study was supported by the National Natural Science Foundation of China [No. 31772499].
